# Microbiome yarns: The Global Phenotype‐Genotype Survey: Episode I: all my worldly goods, including my microbiome, I thee endow

**DOI:** 10.1111/1751-7915.13341

**Published:** 2018-12-04

**Authors:** Kenneth Timmis, Franziska Jebok, Manfred Rohde, Gabriella Molinari

**Affiliations:** ^1^ Institute of Microbiology Technical University Braunschweig Braunschweig Germany; ^2^ Institute for Educational Science University of Freiburg Freiburg Germany; ^3^ Central Facility for Microscopy Helmholtz Centre for Infection Research Braunschweig Germany

*BBZ, Studio 7A, BBZ Plaza, Burbank, 7.30 pm* 1 ^,^ 2 ^,^ 3 ^,^ 4

*Abigail Repor‐Tastory* 5 *, Discovery Presenter, turns to face the camera*:

I, Jack, take you, Jill, to be my wife, to have and to hold from this day forward; for better, for worse, for richer, for poorer, in sickness and in health, to love and to cherish; till death do us part.”……..” With this ring I thee wed, with my body I thee worship, and with all my worldly goods I thee endow. Well, except for those listed in our prenup^6^.

Good evening viewers and welcome to a new episode of ‘Discoveries that Change our Lives’. Our guest this evening is once again Dr. Anastasia Noitall‐Most^5^ from the Streber Elite University of Los Angeles^5^. Good evening Dr. Noitall‐Most *(shaking hands)* and thank you for appearing on the programme.

*Dr. Noitall‐Most:* Good evening Abi; always a pleasure to be here.

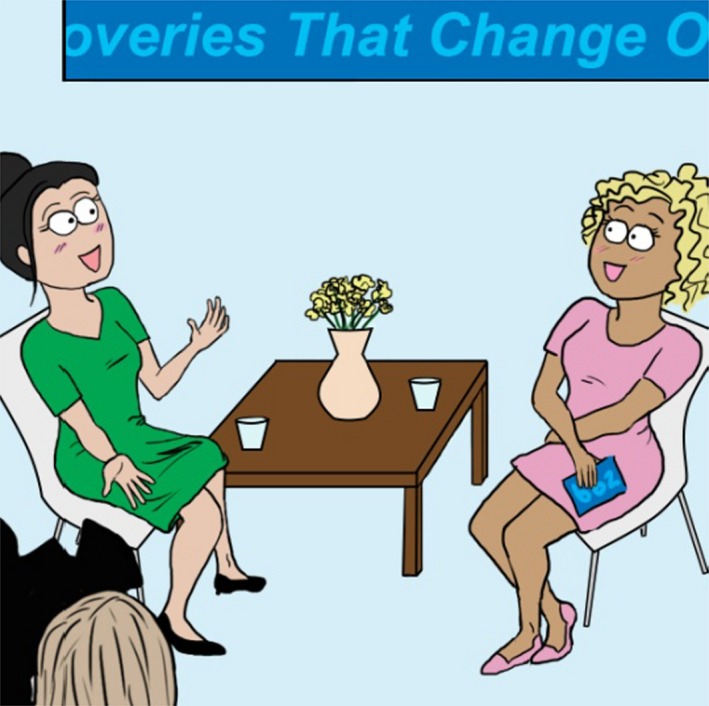

*Ms. Repor‐Tastory*: Ani, this evening we will discuss the results of the most ambitious human microbiome survey to date, and some subsequent trials designed to assess whether or not the correlations it uncovered reflect causalities. AND: we will discuss how couples intending to tie the knot are dealing with the knotty (sorry!) problem of who owns what in terms of their microbiomes.

*Dr. Noitall‐Most:* Yes, Abi: this is indeed a perplexing issue that lawyers are cannily turning into a significant new revenue stream. But before we get into this, we need to inform viewers about recent commercial developments in the field of microbiome science, in order to provide important background.

As many of our viewers will have heard via social media, recent estimates of the number of microbes on and in our bodies suggest that humans are 50% microbial, that is: microbes constitute 50% of all cells that we consist of^7^. And, of course, our microbial half doesn't just sit there and enjoy being ferried around and fed by the other half; it influences and even controls to a significant extent our behaviour, well‐being and health status. In particular, the gastro‐intestinal microbiota, which indeed makes up the major part of the microbial 50%, is centrally involved in immune system development and functioning^8^, and inflammatory responses, including those mediating food sensitivities^9^, has a profound influence on brain activity and mental well‐being, via the so‐called gut:brain axis^10^, and may be involved in the functioning of other organs, via the gut:skin axis^11^, the gut:lung axis^12^, the gut:liver axis^13^, the gut:skeleton axis^14^, etc. This web of modulatory buttons are pushed in part via microbe:host cell interactions, and in part via some of the enormous spectrum of metabolites produced by gut microbes^15^, which are absorbed by the intestine and distributed via the vascular system. Such metabolites may act directly on body processes^16^, or indirectly, by acting as regulatory hormones^17^; indeed, the gut microbiota is now considered to constitute our second endocrine system^17^. In particular, short chain fatty acids produced from our food intake by the gut microbiota seem to play a key role in some processes^16^^,^ 17.

*Ms. Repor‐Tastory*: Yes, Ani: bodily functions we have always considered as peculiarly human, like weight gain tendency, hormonal control and imbalance, mental well‐being, etc., are now being attributed, at least in part, to our microbial friends!

*Dr. Noitall‐Most:* Another pivotal finding is that our microbial 50% varies from individual to individual^18^, so personal differences are to some extent microbially mediated. And, importantly for this evening, a number of things we do during the course of the day (and more so at night, and on birthdays) result in the sharing of bits and pieces of our microbiome with others. So: a key question occupying microbiomologists right now is whether or not our personal characteristics may change as a result of acquiring microbiota from others.

*Ms. Repor‐Tastory*: Yes, Ani: since the start of this programme series about our microbiomes, I often wonder which new microbial friends I am acquiring when lounging in the hot tubs of new acquaintances, drinking a G&T!

*Dr. Noitall‐Most:* Well, you jest, but there have been quite a few publications on infections acquired from hot tubs^19^, some not so pleasant, and there are even diseases called *hot tub lung* and *hot tub folliculitis* 19. Of course, most clean and properly maintained hot tubs do keep down the microbiota we shed into them. BUT, inadequately maintained hot tubs, and indeed other incorrectly managed recreational waters, allow survival and sometimes growth of microbes, including some in the microbiota bounties we shed while relaxing or engaged in sporting activities, and can be a means of microbiota exchange, and of course a source of infections^19^.

*Ms. Repor‐Tastory*: Gosh: I really will have to be more circumspect about accepting late afternoon hot tub G&T invitations in future!

*Dr. Noitall‐Most:* Quite so: new lifestyle fads often come with unanticipated risks! Anyway, to return to the issue of economic and legal aspects of microbiota sharing, we all know by now that a number of clinical conditions caused either by pathogenic microbes or an unhealthy microbial flora, or to use the current technical term *dysbiosis* 20, have been successfully treated by transplants of ‘healthy’ or ‘normal’ microbial communities obtained from donors. The, by now, classical example is the curing by faecal transplants of colitis caused by *Clostridium difficile* 21 Moreover, conditions like obesity^22^ and diabetes^23^ have been linked with specific gut microbial constellations and functionalities, and new correlations are emerging with regularity. These findings have fuelled an explosion of start‐up companies exploring the potential application of microbiota transplants to cure or ameliorate all sorts of conditions.

*Ms. Repor‐Tastory*: Presumably, it won't be long before all animals and plants^24^ of interest and importance to us will be subjected to microbiomological scrutiny, in order to explore transplant opportunities in prevention, therapy and health robustness?

*Dr. Noitall‐Most:* Most certainly! But, to return to humans, in order to be able to treat the growing number of patients, and to enable the growing number of properly controlled trials, there is an enormous demand for carefully characterized and conserved microbiota samples from diverse donors that have been properly collected and stored^25^.

Now while this may seem to be purely a logistical and standardization issue, there exists considerable uncertainty as to what samples are needed. The problems with microbiota samples from donors is that they differ significantly from person to person, and even over time from the same donor, so reproducibility and quality control are challenges, and the potential for transmission of any pathogens in the samples is not negligible. For this reason, there have been great efforts to create simple synthetic microbiota mixes from isolated and characterized individual strains thought to be functionally critical for transplants^26^. However, the big question is: will single strains, or even simple artificial mixes of such strains, be as effective as the highly complex natural microbiota? Until recently, we did not know and it was not unlikely that some conditions may be effectively treated by single strains, and some by synthetic mixes, whereas others would require natural microbiota. For this reason, many transplant companies have been developing all types in parallel.

*Ms. Repor‐Tastory*: Yes, I read somewhere that there is a rapidly growing amount of corporate debt accumulating, as a result of all the transplant cocktails being formulated and trials being undertaken.

*Dr. Noitall‐Most:* Which is a bit of a worry for those of us invested in such companies…..Anyway, this brings us to the issue of *keystone* microbes. A *keystone* is an organism that plays a key role in determining the composition and functioning of an ecosystem or, in our case, the microbiome part of the human biome and how it determines our well‐being^27^ If a particular human disease or characteristic is influenced by a single organism, then in principle its treatment or modification becomes a great deal simpler than those caused by complex microbial constellations. So there is a lot of interest in whether or not *keystone* microbes are involved in any human characteristics.

A second issue is: are natural microbiota, including possible *keystone* microbes, from some donors better than those from others? In other words, will blue blood Adonis Kingman's and Venus Empressa's microbiota contain better microbes, and therefore have a higher intrinsic commercial value, than those from reddish blood Maude Cobbler and Dennis Winterbottom? Of course, we know from biotechnology that some microbial strains are better than others belonging to the same species at producing a particular drug or enzyme that carries out an interesting reaction. So, if this, as seems likely, turns out to be the case for certain microbiota constellations, and especially for *keystones*, this may mean that specific bugs in some people's microbiome may also be superior for the creation of synthetic mixes. So: there is a lot of effort to determine if Adonis’ and Venus’ microbiomes are indeed superior sources of microbes for transplantation than those of Maude and Dennis and, if so, to commercialize them and the relevant individual strains. An amazing number of start‐ups and, especially some of the largest pharma‐food‐personal care‐data processing tech‐partner search companies have formed intricate strategic alliances to develop and market diverse clinical and personal care microbiota products.

*Ms. Repor‐Tastory*: Wow: yes, I always wondered whether blue bloods have inherent superior genes, now also microbiomes, or whether they are like everyone else and just had the luck to be born into a dynasty!

*Dr. Noitall‐Most:* Indeed: I think a quite a few folk have pondered this question, especially the blue bloods themselves! Anyway, as you will recall, to obtain some answers to these and other burning questions, the most ambitious microbiome survey to date, the *Global Phenotype‐Genotype Survey*, was carried out by an international research group assembled by a global consortium of health agencies, in order to confirm existing correlations of influences of microbiota on phenotypes, and to discover new ones. Let's just listen in to the summary of its findings, presented a year ago by Professor Phichit^28^ Dubbelblindangeentee, Coordinator, Global Microbiome Assessment Unit, addressing a session of senior public health officials, directors of microbiome institutes, and heads of diverse NGOs, at the World Biome Health Organisation in Geneva.

*Camera switch from studio to video:*

*Professor Dubbelblindangeentee:* Good morning! It gives me much pleasure to share with you, the policy‐making and decision‐taking international health professionals and agency heads present in the auditorium and viewing via satellite link, the conclusions of the *Global Phenotype‐Genotype Survey*,* GLOPS* for short, a comprehensive worldwide survey on phenotype‐genotype and microbiome individuality and exchange, carried out by a coordinated consortium of essentially all international accredited microbiome centres.

Just to re‐cap: historically, it has been assumed that individual human phenotypes were determined by individual human genotypes, modulated in part by some environmental factors. More recently, however, it has become very evident that the microbiome – the microbial component of the human biome – the microbes inhabiting all of our external surfaces, including the GI tract, the airways and urinary‐genital tract and, occasionally, some internal niches – contributes significantly to human phenotypes. These contributions are additional to the well‐known battery of microbial ecophysiological services to the human host, such as assisting our digestion and releasing and processing nutrients in our food intake, providing a range of essential nutritional requirements we do not make or acquire through our diets, protecting from infection, etc., etc. It is now clear that the microbiome has a pervasive influence on our physical and, importantly, mental health and individual behaviour^29^. The Grand Challenge for microbiomologists, therefore, is to develop strategies and mechanisms to optimize the compositions and functions of our individual microbiomes for maximal health benefits.

Moreover, in addition to the generic contributions the microbiome makes to human biology, it has been found that microbiomes harbour an enormous diversity of microbes, and that everyone's microbiome has a unique composition – qualitatively and quantitatively^30^. A key question this diversity triggers is: to what extent does human individuality result from its microbiome individuality?

The overarching goal of *GLOPS* was to identify which microbes and microbial constellations have the greatest impact on the health and well‐being of human hosts, and how these constellations and their influences are modified by host genetics, lifestyles and practices known or suspected to actively mediate the sharing of microbiomes between individuals. It is the most ambitious survey ever conducted and I hope that its findings will convince you that it brings us to both a better understanding of the relationship between human phenotypes and their microbiomes, of parameters affecting microbiome composition, and how the microbiome may be optimized for better health.

The study was designed to interrogate the following parameters:

- *Phenotype diversity*
- *Genome diversity*
- *Microbiome diversity*
- *Microbiome exchanges*: to what extent are individual differences influenced by *sharing* behaviours?
- *Keystone microbes*: which specific microbes and microbial constellations seem to be keystone in the determination of health‐ and well‐being‐related parameters? and
- *Keystone quality*: among such keystone microbes and microbial constellations, which seem to be particularly good at positively influencing such health parameters?

*GLOPS* involved 173 designated centres in 69 countries around the globe. Each centre recruited on average 1000 subjects, from which multiple, replicate dermal, buccal, faecal and genital samples were collected on 4 occasions, that ultimately yielded 40m microbiota analyses. All centres adopted identical protocols for subject coding, acquisition of subject personal and clinical data, data migration to our central database, patient stratification, microbiome sampling, and DNA extraction‐metagenome sequencing‐sequence analysis.

An aliquot of every sample was stored locally. Once correlations emerged, relevant sample aliquots were immediately couriered to the Imaging Group of Mabriella Golinari and Ranfredy Mohde of the Walpur Gisnacht Institute for Cellular Pathology in Bad Hurzbarg in Northern Germany^31^, which is the leading group worldwide for new microbe isolation and identification, and which was responsible for keystone microbe isolation and characterization.

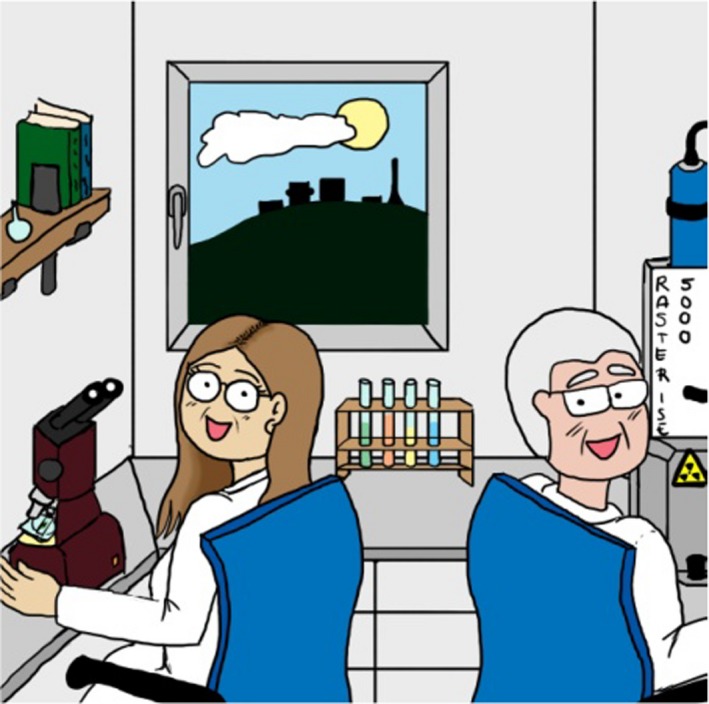

Because this audience contains both specialists and executives, I shall first provide an overall general summary of the results, and then discuss the trial details for the specialists, in order to allow the executives to move on to other pressing issues.

To investigate the influence of sharing of microbiota, a comprehensive spectrum of behavioural parameters were queried, among which were:

- *greetings*, which ranged from no physical contact – raised eyebrow^32^ faint nod and other bodily gestures, vocal greeting, kiss blowing – to physical contact, ranging from handshakes and variations thereof, hugs, 1/2/3 kisses on the cheek/lips/feet, Hongi and variations thereof, etc. (More intimate practices thought to exist in a few cultures were not considered in this study.)
- *sexual sharing* of microbiota, frequency, diversity of partners, body surfaces involved, number of partners in each session, use of physical barriers to conception, use of toys, etc.
- *non‐sexual sharing* microbiota practices, such as regular travel by bus, train, plane, sharing office space, social gatherings such as parties,

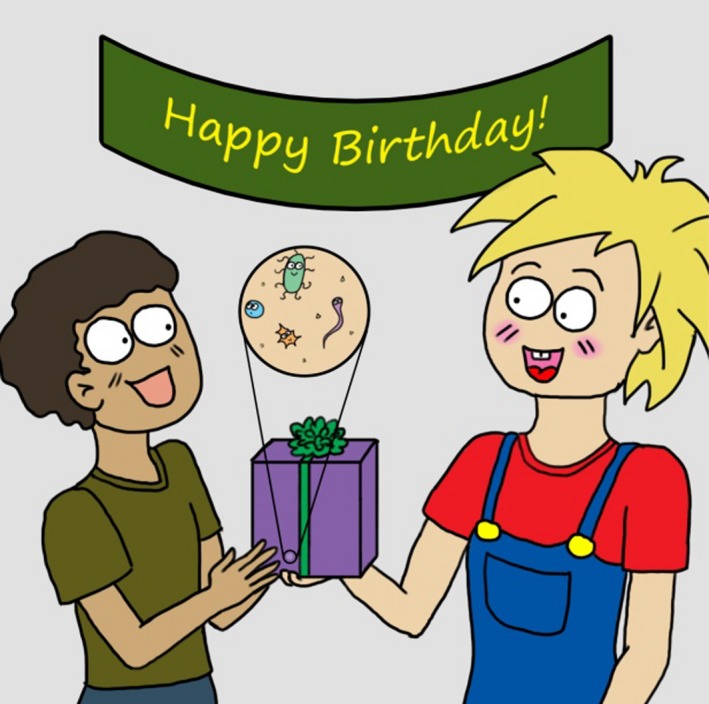

for couples sharing a bed, the degree of body coverage by nightware, if worn, relaxing in hot tubs, personnel caring for incapacitated patients, etc. Potential subjects practicing extreme physical contacts, like Sumo wrestlers, safety‐first teachers regularly practicing mouth‐to‐mouth resuscitation, etc. were excluded from the study.

- *non‐sexual sharing* of microbiota mediated by household pets, using dogs as a proxy. The following categories of canine‐mediated exchange of household microbiota were considered (a) pet kept in a kennel outside, (b) pet allowed in all rooms except kitchen and bedrooms, (c) dog allowed to sleep at the bottom of the bed and lick faces of household members.
- *non‐biologically mediated mechanisms of sharing* of microbiota, categorized as households/places of work being located close to waste treatment plants, densely populated buildings lacking air exhaust filters, farmland frequently treated with pig slurry, parklands regularly manicured with leaf blowers, etc.

The renowned mathematician, Professor Fidget Jones^33^, served in this study as Deputy Coordinator, statistician and modeller, was responsible for the *GLOPS* trial design, defined subject and parameter stratifications, evaluated the results, and developed predictive models. He is with us today and I give the floor to him to summarize the conclusions of the study. Fidget!

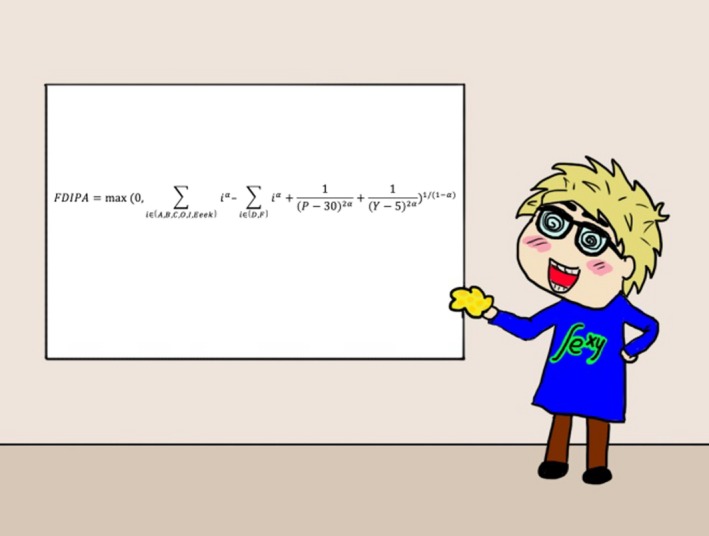

*Professor Jones*: Errrr, thank you Phichit. Well….ummm….given the complexity of the *GLOPS* study, the even greater complexity of its results, the necessity for development of entirely new algorithms to extract the maximum information from the data and model it, and the limited time at my disposal today, I will just summarize the principle findings. These are:

1. every non‐genetically‐identical human has a *unique personal biological phenotype profile* or *UBIP,* which varies significantly among individuals in terms of ecophysiology, appearance, behaviour, personality, health robustness and disease susceptibility, and ageing characteristics, a unique genome, and a unique microbiome.
2. Certain components of the *UBIP* correlate strongly with specific human genome features, and others correlate strongly with specific microbiome features.
3. Specific features of human genomes correlate strongly with specific features of microbiome composition^34^.
4. These findings suggest that the unique genome of each and every one of us determines important elements of our microbiome composition. And, together, our *human* genome and our *microbiome* genomes determine a considerable number of our unique characteristics, of our *UBIP*. In other words: who and what we are the result of the integrated expression of our human and microbiome genes. Put another way: *the metagenome of our biome* largely determines our individual biological phenotypes.
5. Some human characteristics seem to be strongly influenced by individual microbial strains, raising the possibility that there may exist certain *keystone* microbes responsible for such characteristics.

These results are now in press and will appear next month in *Natural Science* 35. Of course, the conclusions I have presented are all tentative, since they are based on correlations, but have allowed me to develop powerful new predictive models that are the basis of the design of several human trials involving microbiota exchanges to establish causalities. These trials are well underway and their results can be expected within the next 12 months.

I should now like to return the floor to Professor Dubbelblindangeentee, who will take us through the subject selection process, the clinical assessments carried out, the specific information queried in the trial, and the all‐important data security measures.

Fade out……fade in Studio 7A.

*Ms. Repor‐Tastory*: Yes: that was absolutely amazing at the time!

*Dr. Noitall‐Most:* Indeed! We knew that our microbiomes influence a number of health‐related aspects but the magnitude and range of the influence on human characteristics suggested by the survey was surprising. And now, one year later, we have the results of the trials carried out to test the predictions of the correlations found in the survey. The trials have established a number of really interesting human characteristics that are significantly influenced by our microbiome, and that are subject to modification by microbiota changes. Amazingly, in some cases, specific individual microbes were shown to act as *keystone* bugs^27^ influencing specific human characteristics, which will significantly accelerate the translation of microbiome advances into medical and commercial exploitation. However, tonight I will only discuss the general issue of microbiota exchanges, and return to the specifics, in particular about which keystone microbes control which phenotypes in future programmes.

*Ms. Repor‐Tastory*: Fine Ani: we will look forward to learning more about these fascinating findings.

*Dr. Noitall‐Most:* So, as we might have anticipated, a key microbiota sharing practice is physical intimacy^36^, so succinctly encapsulated in the phase of the late, great microbiologist Stanley Falkow: *sex is simply the mixing and matching of mucus membranes (which had in recent years become more and more innovative)*, and so succinctly depicted in a cartoon he drew during one of his classes for medical students, to illustrate person‐to‐person disease transmission, shown here on the screen^37^

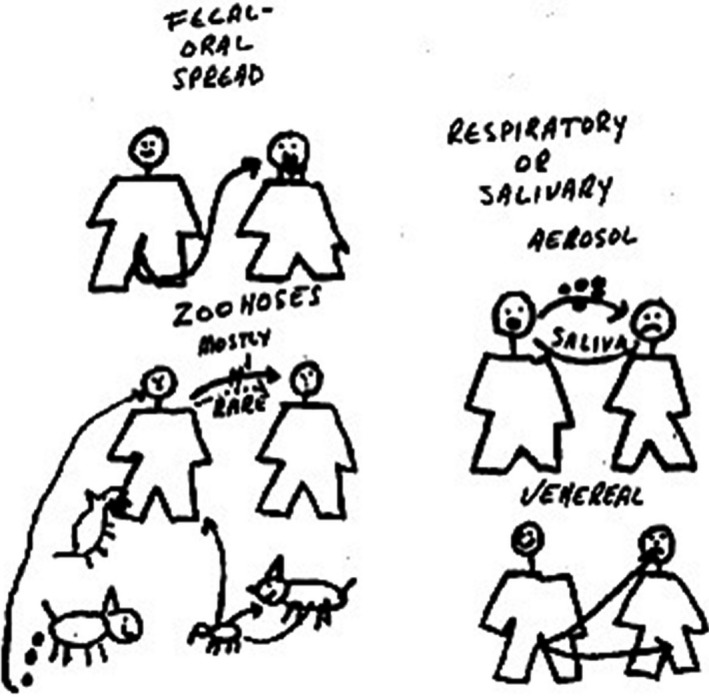

Of course, his famous comment focussed on sexually transmitted infections, but his cartoon encapsulates the modes of exchange of other mucosal microbes and, indeed, non‐mucosal microbes during human interactions, sexual and non‐sexual (e.g. food preparation and sharing, aerosol sharing in confined spaces, pet sharing, etc.). And of course, if microbiota exchanges through such interactions so easily result in a dramatic phenotypic change, in the case of venereal disease, from a healthy state to a rather uncomfortable, not to say somewhat embarrassing, disease state requiring medical attention, it is to be expected that they can mediate other phenotypic changes, not necessarily negative changes.

*Ms. Repor‐Tastory*: Golly: our interactions with one another, and with our pets, are a veritable microbial stock exchange, with some acquisitions enhancing our health wealth, and others causing health crises!

*Dr. Noitall‐Most:* Yes, though unlike the normal variety of stock exchange, we do not get to choose what we receive.

However, Abi: one interesting peripheral finding of the survey was directly relevant to our favourite mode of relaxation. The Bad Hurzbarg Group managed to isolate a bacterium from samples of people with a predilection for G&T‐time in the hot tub, but that was lacking from non‐ or rare‐hot tubbers. This bug has not been seen before and thus a member of the rare biosphere^38^. Interestingly, when given orally to humans that ordinarily do not drink G&T and do not submerge themselves in hot tubs, it induces a sense of well‐being. Mab and Ran did a metabolome study of this bug and found that it produces a chemical resembling a well‐known relaxant.

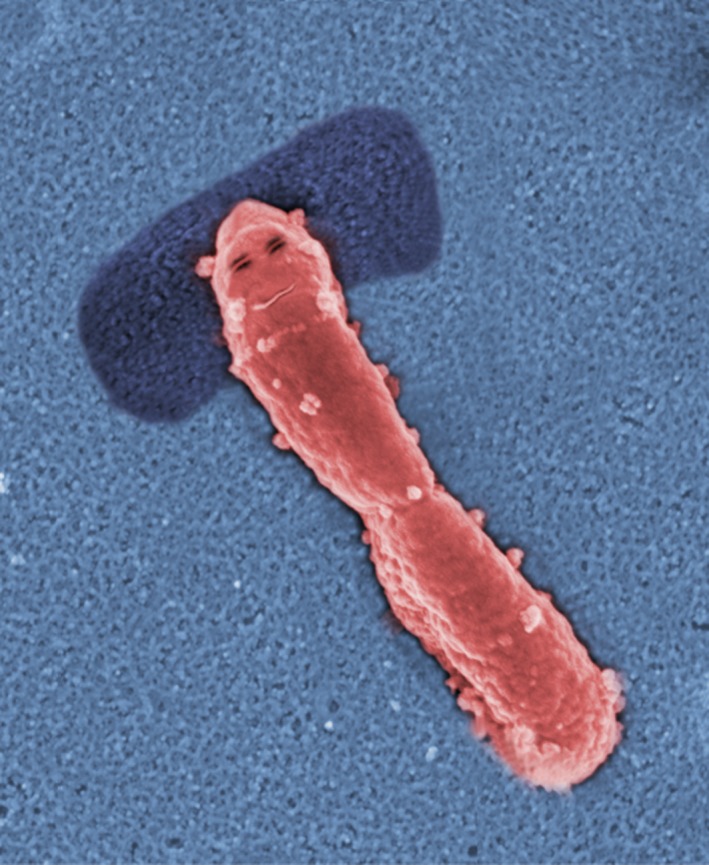

They called this bug *Jacuzzia soporifico* 39 *,* but hot tub users immediately christened it Jacso. Apparently Jacso forms biofilms, which are highly resistant to chlorine disinfectants, around the water:air interfaces of hot tubs where they are orally acquired by G&T‐drinking occupants^40^.

*Ms. Repor‐Tastory, eagerly:* Okay, Ani: but were keystone microbes determining human characteristics identified, and are the microbiomes of Adonis and Venus better than those of Maude and Dennis?

*Dr. Noitall‐Most:* Oh, indeed! But new discoveries concerning phenotype‐influencing microbiota and keystone microbes will have to wait until the next show, because we are running out of time. However, I will give a few teasers to whet your and viewers’ appetites: let us say that our vocal characteristics and several aspects of our personal appearance and behaviour including, dare I say it, libido, are all influenced by our microbiomes.

*Ms. Repor‐Tastory,* with a *can't‐wait‐to‐find‐out* look on her face: oooohh: I think you'll have to give me some advance intelligence over a margarita after the programme!

*Dr. Noitall‐Most:* Of course, Abi! But to answer your question about blue versus red blood: it turned out that the microbiota of aristocrats are, on average, less diverse and less potent at improving the biological properties tested than those of the hoi polloi, despite their astonishingly high frequencies of microbiota sharing activities among themselves. According to Professor Tim Kennis of the Queenton Institute of Advanced Studies, since the diversity of human genomes, and their phenotypic consequences – that is, the physiological differences between us – influence the composition of the resident microbiomes, and since the resident microbiomes superimpose their own physiological modifications, there has been and still is co‐evolution of the two partners of the biome. His explanation is that, because there is a greater level of interbreeding among blue bloods, there is a lower level of human genetic diversity and hence a lower degree of microbiome diversity. As a result, the evolutionary speed of blue blood biomes is slower, and they have less robust microbiomes. Exclusivity comes at a cost. Of course, they have evolved to compensate for the consequential less robust health by taking breeding more seriously, which explains their promiscuity.

*Ms. Repor‐Tastory, looking enlightened, and with increased interest*: Oh: that is the reason! And Maude and Dennis have better microbiomes and better keystones; that is interesting!

*Dr. Noitall‐Most:* Yes, but remember we are talking about average microbiomes: I am quite sure that there are some Adonis and Venus individuals with super microbiomes and super robust constitutions.

*Ms. Repor‐Tastory:* Well, that *is* a relief!

*Dr. Noitall‐Most:* I agree! But anyway, there was one particularly interesting finding relating to the microbiota of aristocrats that I might mention at this juncture. It turns out that the microbiota of the oral cavity of long pedigree aristocrats contains several related microbes hardly found in ordinary folk or in the *nouveau riche*, and which correlate with the posh diction^41^ and sophisticated vocabulary of blue bloods. Mab and Ran were not able to isolate these and study them, so we have no evidence of causality, but they were able to visualize these microbes *in situ*. They were amorphous, non‐descript bacteria belonging to a new Candidate Division tentatively designated *Exclusivia* inhabiting discrete regions of the epiglottis epithelial sheet. They named them *Aristodictionas plummyvowela* strains 1–4.

*Ms. Repor‐Tastory:* What happens when they get transferred to ordinary folk during exploratory kissing?

*Dr. Noitall‐Most:* Interesting question, Abi! It turns out that only one of the *A. plummyvowela* strains has been found in other folk, especially in socially upwardly mobile people, who do articulate in a rather exaggerated manner, as in *Aive just bin down to the showroom and ordered the new series 5 model*.

*Ms. Repor‐Tastory:* Yes, this funny way of speaking was the basis of a number of sitcom comedies in the past.

*Dr. Noitall‐Most:* In any case, there are some very interesting findings about the microbiology of diction from the GLOPS study, which we will deal with in a later programme.

But all of this research is leading to some exciting developments in our understanding of the ramifications of what sort of humans we are and how this is determined by our microbial friends, on one hand, and the commercial applications of this knowledge on the other. The recognition that some of our features are determined or influenced by individual microbes – *keystone* microbes – and that both the *keystone* microbes and the microbiota which play roles in our phenotypes vary in the magnitude of their influence – some are better than others – has led to a number of economic assessments of the commercial values of donor samples. This, in turn, has spawned a plethora of secondary economic activities, such as trading microbiota futures by brokers, insuring own microbiota‐microbiomes by those people in show business who habitually, very publically and for astronomical sums insure their key show assets, holding archived high value microbiota samples as investment instruments, like old masters and fine wines, but also growth in the whole public culture of microbiota applications and its aficionados, and faux‐medical gurus (microbiota transplants have become next generation quinoa, etc.). And of course the topic provides enormous copy for newspapers and magazines, and career advancement for agile reporters and analysts. There is even a growing virtual currency – the mibithaler, or MBT – based on the real donation of samples for archiving, their virtual valuation, and use of the virtual values for trading and saving.

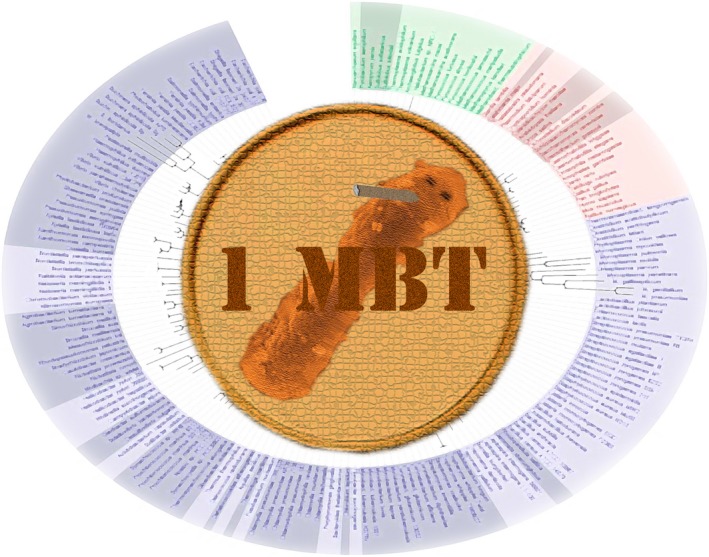

*Ms. Repor‐Tastory:* Well, I never! the financial ramifications of the microbiome world are evolving at an impossible speed. I heard that a number of companies specializing in *wellness* products are aggressively marketing transplant products of all sorts. Who on earth can control the microbiome industry and ensure that microbiome technology is exploited safely?

*Dr. Noitall‐Most:* Yes, Abi: there is, as you might expect, a whole raft of legal, ethical and cultural issues and uncertainties emerging from the business of microbiota transplantation. One example is that some cultures, while accepting the existence of the microbiome, do not entertain its influence on the human entity – they believe that all human characteristics are inherent – and have banned the use and sale of transplant materials. Of course, this has spawned a black market in such products controlled by black sheep. But of course, the illicit trade in transplant samples is, like buying drugs on the internet, fraught with risks of provenance and quality. Indeed, some samples have been shown to consist only of a poor quality flour. There are some black sheep out there^42^!

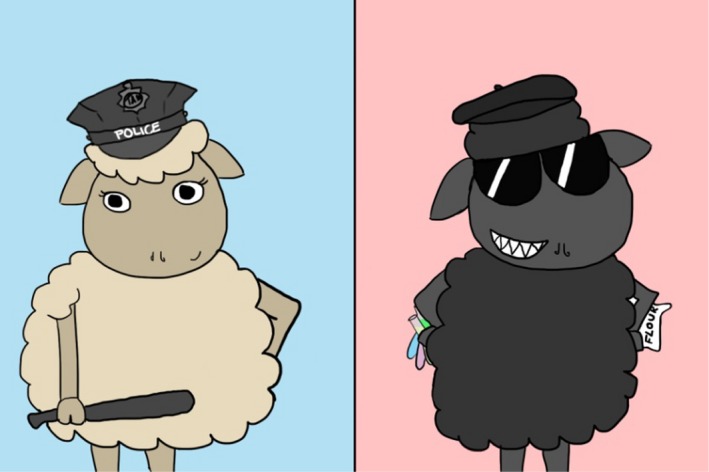

*Ms. Repor‐Tastory:* How interesting! And I recently read somewhere that some philanthropic folk are donating while alive, or willing after death, their microbiota samples to charities and other voluntary services.

*Dr. Noitall‐Most:* Yes, Abi, and this brings us nicely to the knotty issue of who owns, or at least shares with the partner, what? One central component in the marriage vows of traditional weddings is the commitment of *all my worldly goods I thee endow*. This literally means the gifting to the other partner of all personal resources. However, in modern weddings, the prenup has taken centre stage because the average 5‐year half‐life of marriages which precedes the inevitable 7‐year itch, has had a sobering influence on the perception of the partneristic future. Previously, prenups were mostly concerned with financial parameters of the involved partners, and the biological baggage (children from previous partners ferried into the new relationship). But now, instead of the primary consideration being concerned with the past, prenups are increasingly concerned with the future, in particular to potential subsequent partner(s) and the evolution of such relationships.

So, for example, does the *I thee endow* vow include eggs of the female partner frozen, while pursuing a career, for potential future fertilization, and sperm of the male partner frozen to provide last gasp fertilization options to perpetuate their lines?

*Ms. Repor‐Tastory:* Gosh, yes: a central pillar of marriage is the sharing eggs and sperms, so decisions about what is permitted after a divorce with seed frozen during a marriage is indeed a perplexing issue!

*Dr. Noitall‐Most:* Exactly! But: the topic that concerns us today is the increasing trend of young people to store microbiota samples e.g., faecal microbiota, for possible transplants at a later date, either for personal use or use on others. So the issue is one of ownership and whether or not the microbiomes become part of the *goods I thee endow*. Moreover, since all microbiomes are not equal and the ‘better’ ones have significant value, both to the other partner and as tangible commodities, things have gone a step further and couples are attempting to assess the value – personal and/or economic – of the partner's microbiome, prior to tying the knot. However, superimposed on all of this is the fundamental fact that marriage precipitates comprehensive microbiota exchanges anyway, so the issue of *goods I thee endow* is according to some, entirely irrelevant with regard to the microbiome.

And then, of course, there is the uncomfortable issue of infidelity which, in terms of microbiome sharing between married couples, means the possibly unaware and almost certainly unwanted sharing of third party microbiota.

*Ms. Repor‐Tastory:* Golly: that is a thought – even worse than the hot tub G&T sharing experience!

*Dr. Noitall‐Most:* Absolutely! Of course all of this is a sort of amusing topic for young couples thinking about the great experiment of marriage, and may result in light‐hearted prenups.

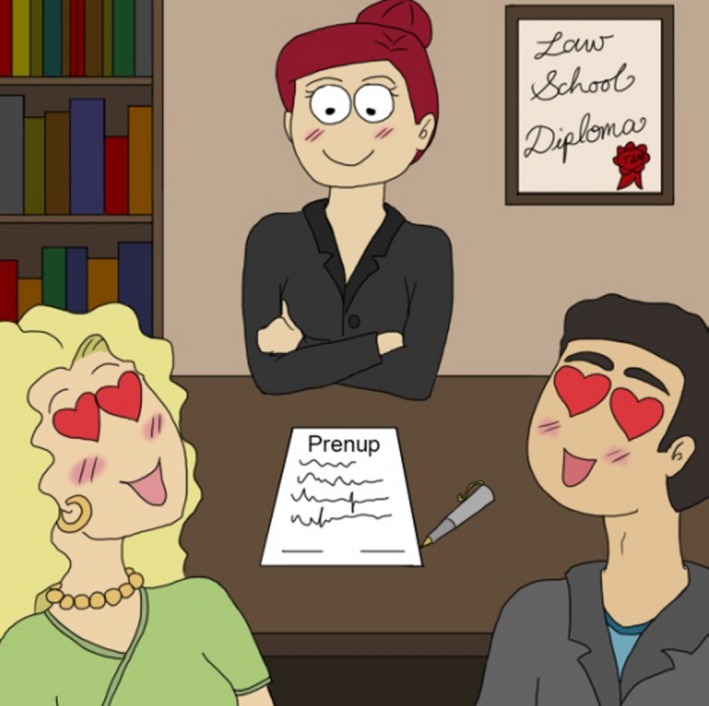

Divorce is, however, a different matter and eggs, sperms and microbiota samples become settlement assets for lawyers to negotiate. So there is a vigorous discussion taking place about modernizing the wedding vows to incorporate the microbiome element.

While the whole business of marriage vows is currently in a state of flux, there are several that are increasingly adopted. These include:

*A*, will you take *B* to be your wife?
Will you love and comfort, honour and protect,
and, forsaking all others,
be faithful as long as you both shall live?
To have and to hold,
From this day forward,
For better for worse,
For richer for poorer,
In sickness and in health,
To love and to cherish,
To freely and selflessly share your reproductive seeds and microbiomes,
So long as ye shall consider your partnership to have a future,
After which ye shall consult a lawyer to divide in an equitable manner all common assets.

The last line of this was, of course, written by a lawyer, since, though legally logical, it is obvious to any microbiologist that equitably dividing microbiomes is an absolutely impossible task, thus providing a cause for endless litigation, and the ultimate transfer of all contested assets to the lawyer.

*Ms. Repor‐Tastory:* Gosh, Ani: hasn't the world become philistine in recent years?

*Dr. Noitall‐Most:* Yes, Abi, for sure! but here's the thing regarding marriage and microbiomes: if I am a biome, consisting of a human and my integral microbiome, then by definition the microbiome is a key stakeholder in all actions that influence the future well‐being of the biome. Then: is it at all reasonable that the human part of me unilaterally undertakes a serious commitment like marriage, which involves long(ish) intimate physical interactions with another biome, or should marriage be a biome:biome contract? And, if so, how should my microbiome be consulted on the issue or, at least, its compatibility with the ‘other’ microbiome assessed?

*Ms. Repor‐Tastory:* Golly: I had not thought about that! It certainly bears thinking about. In the meantime, let us return to the traditional ending to our programmes: are there important applications of the discoveries we learned about this evening?

*Dr. Noitall‐Most:* Most certainly! Quite apart from the non‐microbiomological applications we have touched on this evening, relating to legal aspects of ownership and inheritance, there are some exciting new biomedical applications currently being explored. One is very exciting work initiated by Professor Vic Torde, Head of the Lorenzo von Syntech High Security Institute for Artificial Life in Madrid, who is applying synthetic microbiology to reconfigure an important but so far confidential keystone bacterium identified in the *GLOPS* study, which he calls *Synkey*. He has two goals, the first of which is to design a very competitive version of *Synkey* able to establish and maintain itself long‐term in the gut, and that in addition to its normal keystone function, will serve as a therapy delivery system for different metabolites that combat diseases or increase well‐being.

*Ms. Repor‐Tastory:* Gosh: so the day of synthetic microbes as therapies is drawing close!

*Dr. Noitall‐Most:* Most certainly! Vic's second goal is even more ambitious. Thus far, attempts to restore normality to patients suffering from disease resulting from dysbiosis involve microbiota transplants ranging from faecal material to less complex mixtures and even single microbes^26^. The problem is that complex mixtures may be associated with significant risks and, in any case, suffer from the challenges of standardization, reproducibility and quality control, whereas single strains are often ineffective at correcting dysbiosis situations. What Vic is doing is to use the camel nanobody display approach^43^ to evolve a living scaffold for the precision assembly of microbial consortia of an exact, predetermined composition. In short, camel antibodies that specifically bind to ligands/antigens present on the surface of selected gut microbes essential for restoring normality in a dysbiosis situation are displayed on the surface of *Synkey*. Mixing *Synkey* with partner microbes displaying the appropriate natural or engineered ligands/antigens results in a sort of click‐fastener joining and the formation of physically linked partners in a precisely configured consortium, designed to treat a specific dysbiosis^44^. And in collaboration with immunologist partners, his group is designing *Synkey* variants displaying nanobodies specific for host cells, such as the gut‐associated lymphoid tissue, in order to target *Synkey* delivery systems to such tissues where they can precisely stimulate desired immune responses^44^.

*Ms. Repor‐Tastory:* My goodness, whatever will synthetic microbiology deliver next?

*Dr. Noitall‐Most:* Aahhh: synthetic microbiologists are indeed creative creatures! Another important application is the explosion in demand for genome‐metagenome compatibility profiling in partner searches. A number of partner search companies have teamed up with genome sequencing‐storage‐analysis companies and offer phenotype‐genotype profiling as part of their services. Some even go as far as offering compatibility assessment analyses/scores of offered partner options, but received wisdom is that these are next to useless, since most relationships are based on emotional chemistry which is, so far, not reliably interrogated through metagenomics.

*Ms. Repor‐Tastory:* Yes, I cannot imagine offering my metagenome data to strangers, even a highly abstracted form, and anyway, a candlelit dinner in a small, discrete and preferably exclusive restaurant almost always enables a good assessment of partnership potential.

*Dr. Noitall‐Most:* And that, of course, is a key issue – the one of security of such personal information, so that is another major area of application, namely the development of new encryption systems and firewalls for metagenomic data storage facilities, which is occupying quite a few microbial informaticians.

*Ms. Repor‐Tastory:* well, viewers: on that note we end this edition of ‘Discoveries that Change our Lives’. We will be back next week with a follow‐up edition to reveal some more of the amazing findings of the GLOPS study and its experimental follow‐up.

*As the camera pans away, viewers see Abi leaning towards Ani and saying: now what was that about libido and…*

++++++++

## Conflict of interest

None declared.

## Explanatory Notes for Online Version at www.theabsurdmicrobe.com

## Footnotes

1 To view previous issues of *Microbiome Discoveries that Change our Lives*, go to http://www.theabsurdmicrobe.com/discoveries-oldstuff

2 Health Warning. This article is a figment of the authors’ imagination; any serious contemplation may unnecessarily challenge the mental harmony of the reader.

3 any possible resemblance of the fictional characters and organisations in this fable to a real person, organisation or enterprise is coincidental and unintentional

4 Dedicated to pioneering researchers of the microbiome and especially those revealing human phenotypes influenced by our microbial partners, to pioneering synthetic microbiologists, to the memory of Stanley Falkow, who pioneered the field of microbial pathogenesis and cellular microbiology (see, e.g. ref. 38), Dan Koshland, Jr., father of Dr. Noitall (e.g. see Editorial: A simple proposal for health care rationing. *Science* **264**: 1515), and David Jones, father of Daedalus, who invented Dreadco (see, e.g. https://www.newscientist.com/article/mg23531413-500-what-did-daedalus-talk-about-in-septembers-past/; https://www.improbable.com/2017/07/27/sad-news-daedalus-david-jones-is-gone/), science humourists all

5 http://www.theabsurdmicrobe.com/discoveries/

6 Prenup: https://legal-dictionary.thefreedictionary.com/Prenup

7 Sender, R., Fuchs, S., and Milo, R. (2016) Are we really vastly outnumbered? Revisiting the ratio of bacterial to host cells in humans. *Cell* **164:** 337–340.

8 Round, J.L., and Mazmanian, S.K. (2009). The gut microbiome shapes intestinal immune responses during health and disease. *Nature Revs Immunol* **9:** 313–323; Martin, R., Nauta, A.J., Ben Amor, K., Knippels, L.M.J., Knol, J., and Garssen, J. (2010) Early life: gut microbiota and immune development in infancy. *Beneficial Microb* **1:** 367–382; Maslowski, K.M. and Mackay, C. R. (2011) Diet, gut microbiota and immune responses*. Nat Immunol*. **12**: 5–9; Prince, B.T., Mandel, M.J., Nadeau. K. and Singh, A.M. (2015) Gut microbiome and the development of food allergy and allergic disease. *Pediatr Clin North Am*. **62**: 1479–1492; Gomez de Agüero, M., Ganal‐Vonarburg, S.C., Fuhrer, T., Fuhrer, T., Rupp, S., Uchimura, Y., Li, H., et al. (2016) The maternal microbiota drives early postnatal innate immune development. *Science* **351**: 1296–1302.

9 Man, S.M. (2018) Inflammasomes in the gastrointestinal tract: infection, cancer and gut microbiota homeostasis. *Nat Rev Gastroenterol Hepatol*. https://doi.org/10.1038/s41575-018-0054-1; Caminero, A., Meisel, M., Jabri, B., and Verdu, E.F. (2018) Mechanisms by which gut microorganisms influence food sensitivities. *Nat Rev Gastroenterol Hepatol*. https://doi.org/10.1038/s41575-018-0064-z; Stoll, M.L., Pierce, M.K., Watkins, J.A., Zhang, M., Weiss, P.F., Weiss, J.E., et al. (2018) *Akkermansia muciniphila* is permissive to arthritis in the K/BxN mouse model of arthritis *Genes Immun, in press*.

10 Hsiao, E.Y., McBride, S.W., Hsien, S., Sharon, G., Hyde, E.R., McCue, T., et al. (2013) Microbiota modulate behavioral and physiological abnormalities associated with neurodevelopmental disorders. *Cell* **155**: 1451–1463; Sherman, M.P., Zaghouani, H., and Niklas, V. (2015) Gut microbiota, the immune system, and diet influence the neonatal gut‐brain axis*. Pediatr Res* **77**: 127–135; https://www.ted.com/talks/heribert_watzke_the_brain_in_your_gut; Sampson, T.R. and Mazmanian S.K. (2015) Control of brain development, function, and behavior by the microbiome. *Cell Host Microbe* **17**: 565–576; Dinan, T.G. and Cryan, J.F. (2017) Gut‐brain axis in 2016: brain‐gut‐microbiota axis – mood, metabolism and behaviour. *Nat Rev Gastroenterol Hepatol* **14**: 69–70; Burokas, A., Arboleya, S., Moloney, R.D., Peterson, V.L., Murphy, K., Clarke, G., et al. (2017) Targeting the microbiota‐gut‐brain axis: prebiotics have anxiolytic and antidepressant‐like effects and reverse the impact of chronic stress in mice. *Biol Psychiatry* **. 82**: 472–487; Hoban, A.E., Stilling, R.M., Moloney, G., Shanahan, F., Dinan, T.G., Clarke, G., et al. (2017) The microbiome regulates amygdala‐dependent fear recall. *Mol Psychiatry* **23**: 1134–1144; Vitetta, L., Vitetta, G., and Hall, S. (2018) The brain‐intestinal mucosa‐appendix‐ microbiome‐brain loop. *Diseases* **6**. pii: E23. https://doi.org/10.3390/diseases6020023; Horne, R. and Foster, J.A. (2018) Metabolic and microbiota measures as peripheral biomarkers in major depressive disorder. *Front Psychiatry* https://doi.org/10.3389/fpsyt.2018.00513

11 Lee, S.‐Y., Lee, E., Park, Y.M., and Hong, S.‐J. (2018) Microbiome in the gut‐skin axis in atopic dermatitis. *Allergy Asthma Immunol Res* **10:** 354–362; Salem, I., Ramser, A., Isham, N., and Ghannoum, M.A. (2018) The gut microbiome as a major regulator of the gut‐skin axis. *Front Microbiol* **9:** 1459. https://doi.org/10.3389/fmicb.2018.01459

12 Bingula, R., Filaire, M., Radosevic‐Robin, N., Bey, M., Berthon, J.Y., Bernalier‐Donadille, A., et al. (2017) Desired turbulence? Gut‐lung axis, immunity, and lung cancer. *J Oncol* 2017;**2017**:5035371. https://doi.org/10.1155/2017/5035371.

13 Liu, H. X., Hu, Y., and Wan, Y.J. (2016) Microbiota and bile acid profiles in retinoic acid‐primed mice that exhibit accelerated liver regeneration*. Oncotarget* **7**:1096–1106; Neyrinck, A. M., Taminiau, B., Walgrave, H., Daube, G., Cani, P.D., Bindels, L.B., et al. (2017) *Spirulina* protects against hepatic inflammation in aging: an effect related to the modulation of the gut microbiota? *Nutrients* **9:** pii: E633. https://doi.org/10.3390/nu9060633; Beaumont, M., Neyrinck, A.M., Olivares, M., Rodriguez, J., de Rocca, Serra.A., Roumain, M., et al. (2018) The gut microbiota metabolite indole alleviates liver inflammation in mice. *FASEB J* https://doi.org/10.1096/fj.201800544

14 Sjögren, K., Engdahl, C., Henning, P., Lerner, U.H., Tremaroli, V., Lagerquist, M.K., et al. (2012) The gut microbiota regulates bone mass in mice. *J Bone Min Res **27***: 1357–1367; McCabe, L., Britton, R.A. and Parameswaran, N. (2015) Prebiotic and probiotic regulation of bone health: role of the intestine and its microbiome. *Curr Osteoporos Rep* **13**: 363–371; Charles, J.F., Ermann, J., & Aliprantis, A.O. (2015) The intestinal microbiome and skeletal fitness: connecting bugs and bones. *Clin Immunol* **159**: 163–169; Yan, J., and Charles, J.F. (2017) Gut microbiome and bone: to build, destroy, or both*? Curr Osteoporos Rep* **15**: 376–384; Zhang, J., Lu, Y., Wang, Y., Ren, X., and Han, J. (2018) The impact of the intestinal microbiome on bone health. *Intract Rare Dis Res 7*, 148–155.

15 De Vadder, F., Kovatcheva‐Datchary, P., Goncalves, D., Vinera, J., Zitoun, C., Duchampt, A., et al. (2014) Microbiota‐generated metabolites promote metabolic benefits via gut‐brain neural circuits. *Cell* **156**: 84–96.

16 e.g. Roager, H.M. and Licht, T.R. (2018) Microbial tryptophan metabolites in health and disease. *Nat Comm* https://doi.org/10.1038/s41467-018-05470-4, and references therein.

17 Clarke, G., Stilling, R.M., Kennedy, P.J., Stanton, C., Cryan, J.F., and Dinan, T.G. (2014) Gut microbiota: the neglected endocrine organ. *Mol Endocrinol* **28,** 1221–1238; Brown, J.M., and Hazen, S.L. (2015) The gut microbial endocrine organ: bacterially derived signals driving cardiometabolic diseases. *Annu Rev Med* **66:** 343–359; Koh, A., De Vadder, F., Kovatcheva‐Datchary, P. & Bäckhed, F. (2016) From dietary fiber to host physiology: short‐chain fatty acids as key bacterial metabolites. *Cell* **165:** 1332–1345; Louis, P. and Flint, H.J. (2017) Formation of propionate and butyrate by the human colonic microbiota. *Environ Microbiol* **19**: 29–41.

18 Sender, R., Fuchs, S., and Milo, R. (2016) Are we really vastly outnumbered? Revisiting the ratio of bacterial to host cells in humans. *Cell* **164:** 337–340;

19 Zacherle, B.J., and Silver, D.S. (1982) Hot tub folliculitis: a clinical syndrome. *West J Med* **137**: 191–194; Rickman, O.B., Ryu, J.H., Fidler, M.E., and Kalra, S. (2002) Hypersensitivity pneumonitis associated with *Mycobacterium avium* complex and hot tub use. *Mayo Clin Proc* **77**: 1233–1237; Crnich, C.J., Gordon, B., and Andes, D. (2003) Hot tub‐associated necrotizing pneumonia due to *Pseudomonas aeruginosa*. *Clin Infect Dis* **36**: 55–57; Yu, Y., Cheng, A.S., Wang, L., Dunne, W.M., and Bayliss, S.J. (2007) Hot tub folliculitis or hot hand‐foot syndrome caused by *Pseudomonas aeruginosa*. *J Am Acad Dermatol* **57**: 596–600; Fjällbrant, H., Akerstrom, M., Svensson, E. and Andersson, E. (2013) Hot tub lung: an occupational hazard. *Europ Respiratory Rev* **22**: 88–90; Hlavsa, M.C., Roberts, V.A., Kahler, A.M., E.D. Hilborn, T.R. Mecher, M. J. Beach, et al. (2015) Outbreaks of illness associated with recreational water–United States, 2011‐2012. *Morb Mortal Wkly Rep* **64:** 668–672; https://www.cdc.gov/healthywater/swimming/swimmers/rwi.html

20 Brüssow, H. (2016) Biome engineering‐2020. *Micro. Biotechnol* **9**: 553–563.

21 Eiseman, B., Silen, W., Bascom, G.S., and Kauvar, A.J. (1958) Fecal enema as an adjunct in the treatment of pseudomembranous enterocolitis. *Surgery* **44**: 854–859; Seal, D.V., Borriello, S.P., and Piper, M. (1989) Bacteriotherapy for *Clostridium difficile* diarrhea. *Lancet* **1**: 558; Tvede, M. and Rask‐Madsen, J. (1989) Bacteriotherapy for chronic relapsing *Clostridium difficile* diarrhoea in six patients. *Lancet* **1:** 1156–1160; Jorup‐Rönström, C., Håkanson, A., Sandell, S., Edvinsson, O., Midtvedt, T., Persson, A.K., and Norin, E. (2012) Fecal transplant against relapsing *Clostridium difficile*‐associated diarrhea in 32 patients. *Scand J Gastroenterol* **47**: 548–552; van Nood, E., Vrieze, A., Nieuwdorp, M., Fuentes, S., Zoetendal, E.G., de Vos, W.M., *et al*. (2013) Duodenal infusion of donor feces for recurrent *Clostridium difficile*. *N Engl J Med* **368**: 407–415; Rossen, N.G., MacDonald, J.K., de Vries, E.M., D'Haens, G.R., de Vos, W.M., Zoetendal, E.G., and Ponsioen, C.Y. (2015) Fecal microbiota transplantation as novel therapy in gastroenterology: a systematic review. *World J Gastroenterol* **21**: 5359–5371.

22 Bäckhed, F., Ding, H., Wang, T., Hooper, L.V., Koh, G.Y., Nagy, A., et al. (2004) The gut microbiota as an environmental factor that regulates fat storage. *Proc Natl Acad Sci USA* **101**: 15718–15723; Turnbaugh, P. J., Ley, R. E., Mahowald, M.A., Magrini V., Mardis, E.R. and Gordon, J.I. (2006) An obesity‐associated gut microbiome with increased capacity for energy harvest. *Nature* 444: 1027–1031; Turnbaugh, P.J., Backhed, F., Fulton, L., and Gordon, J.I. (2008) Diet‐induced obesity is linked to marked but reversible alterations in the mouse distal gut microbiome. *Cell Host Microbe*. **3**: 213–223; Truax, A.D., Chen, L., Tam, J.W., Guo, H., Koblansky, A.A., Chou, W.C., et al. (2018) The Inhibitory Innate Immune Sensor NLRP12 maintains a threshold against obesity by regulating gut microbiota homeostasis. *Cell Host Microbe*. 24: 364–378.

23 Udayappan, S., Manneras‐Holm, L., Chaplin‐Scott, A., Belzer, C., Herrema, H., Dallinga‐Thie, G.M., et al. (2016) Oral treatment with *Eubacterium hallii* improves insulin sensitivity in db/db mice. *Biofilms and Microbiomes* **2:** https://doi.org/10.1038/npjbiofilms.2016.9; Vatanen, T., Franzosa, E. A., Schwager, R., Tripathi, S., Arthur, T.D., Vehik, K., et al. (2018) The human gut microbiome in early‐onset type 1 diabetes from the TEDDY study*. Nature* **562:** 589–594.

24 Singh, B. K. and Trivedi, P. (2017) Microbiome and the future for food and nutrient security. *Microbiol. Biotechnol* **10**: 50–53; Mitter, B., Pfaffenbichler, N. and Sessitsch, A. (2016) Plant–microbe partnerships in 2020. *Microbiol. Biotechnol* **9**: 635–640.

25 Bojanova, D.P., and Bordenstein, S.R. (2016) Fecal transplants: what is being transferred? *PLoS Biol* **14:** e1002503; van der Lelie, D., Teghavi, S., Henry, C. and Gilbert, J. A.2017. The microbiome as a source of new enterprises and job creation: considering clinical faecal and synthetic microbiome transplants and therapeutic regulation. *Microbiol. Biotechnol* **10**: 4–5; https://medium.com/neodotlife/the-latest-biotech-growth-market-might-be-crap-4bfcdee22e83

26 de Vos, W.M. (2013) Fame and future of faecal transplantations–developing next‐generation therapies with synthetic microbiomes. *Microb Biotechnol* **6**: 316–325.

27 The notion of the *keystone* species, originally proposed by Paine to describe the member of an organism network having a disproportionate positive influence on ecosystem stability, has over the years, evolved in what some might term a chaotic and confusing manner (for a potted history of subsequent definitions and *quasi* alternative terms, see Cottee‐Jones and Whittaker, 2012). One attempt to create a consensus definition, proposed that a *keystone* species is a species whose impacts on its community or ecosystem are large, and much larger than would be expected from its abundance” (Power & Mills 1995:184; see also Herren and McMahon). In this sense, it may be analogous to a node in a network. For many microbiologists, and in the Yarns, *keystone* is used to designate a microbial taxon having ability to nucleate, structure and stabilize a microbial community characterized by a particular composition exhibiting one or more specific functionalities. In other words, the *keystone* microbe may have a deterministic character for a function under study. Paine, R.T. (1969) A note on trophic complexity and community stability. *Am Nat* **103**: 91–93; Mills, L.S., Soulé, M.E., and Doak, D.F. (1993) The keystone‐species concept in ecology and conservation. *BioScience* **43**: 219–224; Paine, R.T. (1995) A conversation on refining the concept of keystone species. *Conserv Biol* **9**: 962–964; Cottee‐Jones, H. W, & Whittaker, R. J. (2012). perspective: The keystone species concept: a critical appraisal. *Front Biogeog* **4**: 117–127; Trosvik, P., and de Muinck, E.J. (2015) Ecology of bacteria in the human gastrointestinal tract—identification of keystone and foundation taxa. *Microbiome* **3**: 44; Berry, D., and Widder, S. (2014) Deciphering microbial interactions and detecting keystone species with co‐occurrence networks. *Front Microbiol* **5**: 219; Agler, M.T., Ruhe, J., Kroll, S., Morhenn, C., Kim, S.‐T., Weigel, D., and Kemen, E.M. (2016) Microbial hub taxa link host and abiotic factors to plant microbiome variation. *PLoS Biol* **14**: e1002352; Herren, C.M., and McMahon, K.D.(2017) Cohesion: a method for quantifying the connectivity of microbial communities. *ISME J* **11**: 2426–2438; Herren, C. M. and McMahon, K. D. (2018), Keystone taxa predict compositional change in microbial communities. *Environ Microbiol* **20**: 2207–2217. https://doi.org/10.1111/1462-2920.14257;.

28 Phichit: Thai boy name for winner

29 Sharma, A. and Gilbert, J. A. (2018) Microbial exposure and human health. *Curr Opin Microbiol* **44**: 79–87.

30 Costello, E. K. Lauber, C.L., Hamady, M., Fierer, N., Gordon, J.I., and Knight, R. (2009) Bacterial community variation in human body habitats across space and time. *Science* **326**: 1694–1697; Rajilić‐Stojanović, M., Heilig, H. G. H. J., Tims, S., Zoetendal, E. G. & de Vos, W. M. 2012. Long‐term monitoring of the human intestinal microbiota composition. *Environ Microbiol* **15**: 1146–1159.

31 see Timmis, K., Jebok, F., Molinari, G., Rohde, M. and Timmis, J. K. 2017. Microbiome yarns: microbiome basis of memory. *Microbiol. Biotechnol* **10:** 1283–1292.

32 e.g. the Mallorquin greeting of vaguest nods, coupled with a faintly raised eyebrow, and hushed utterance of *UEP* (personal communication of Dr. Rafael Bosch Zaragoza, Universitat de les Illes Balears),

33 see Timmis, K., Jebok, F., Rigat, F., de Vos, W. M. and Timmis, J. K. 2017. Microbiome yarns: microbiomology of curly and straight hair. *Microbiol. Biotechnol* **10:** 231–237

34 Goodrich, J. K., Waters, J. L., Poole, A. C., Sutter, J.L., Koren, O., Blekhman, R., et al. (2014) Human genetics shape the gut microbiome. *Cell* **159:** 789–799; Blekhman, R., Goodrich, J.K., Huang, K., Sun, Q., Bukowski, R., Bell, J.T., et al. (2015) Host genetic variation impacts microbiome composition across human body sites. *Genome Biol* **16:** 191. https://doi.org/10.1186/s13059-015-0759-1; Bonder, M. J., Kurilshikov, A., Tigchelaar, E. F., Mujagic, Z., Imhann, F., Vila, A.V., et al. (2016) The effect of host genetics on the gut microbiome. *Nat Genet* **48:** 1407–1412; Abdul‐Aziz, M. A., Cooper, A., and Weyrich, L. S. 2016. Exploring Relationships between Host Genome and Microbiome: New Insights from Genome‐Wide Association Studies. *Front Microbiol* **7:** 1611; Kolde, R., Franzosa, E.A., Rahnavard, G., Hall, A.B., Vlamakis, H., Stevens, C., et al. (2018) Host genetic variation and its microbiome interactions within the Human Microbiome Project. *Genome Med* **10:** 6. https://doi.org/10.1186/s13073-018-0515-8

35 special issue of Natural Science consisting of only 1 article composed of 2 pages of results summary and conclusions, 43 pages of authors and acknowledgements, and 7,658,792 pages of online supplementary information.

36 Kort, R., Caspers, M., van de Graaf, A., van Egmond, W., Keijser, B., Roeselers, G. (2014) Shaping the oral microbiota through intimate kissing. *Microbiome* **2**: 41; Carr, E. J., Dooley, J., Garcia‐Perez, J. E., Lagou, V., Lee, J.C., Wouters, C., et al. (2016) The cellular composition of the human immune system is shaped by age and cohabitation. *Nature Immunol* 17: 461–468; Ross, A. A., Doxey, A. C., & Neufeld, J. D. (2017) The skin microbiome of cohabiting couples. *mSystems 2*(4), e00043–17. https://doi.org/10.1128/msystems.00043-17; Münger, E., Montiel‐Castro, A. J., Langhans, W., and Pacheco‐Lopez. (2018) Reciprocal Interactions between gut microbiota and host social behavior. *Front Integr Neurosci*,** 12** June https://doi.org/10.3389/fnint.2018.00021

37 Kindly provided and reproduced with the permission of Stanley Falkow who, sadly, passed away this year (see, e.g. Cabello, F. C., Cohen, S. N., Curtiss, III, R., Dougan, G., van Embden, J., Finlay, B.B., et al. (2018) Farewell stan. Stanley Falkow: 1934‐2018. *Environm Microbiol* **20**: 2322–2333

38 Sogin, M. L., Morrison, H. G., Huber, J. A., Mark Welch, D., Huse, S.M., Neal, P.R., et al. (2006). Microbial diversity in the deep sea and the underexplored “rare biosphere”. *Proc Natl Aacad Sci USA* **103**: 12115–12120; Jousset, A., Bienhold, C., Chatzinotas, A., Gallien, L., Gobet, A., Kurm, V., et al. (2017). Where less may be more: how the rare biosphere pulls ecosystems strings**.** *ISME J* 2017; 11(4): 853–862.

39 No, sorry: this is not Jacso but rather *Pseudomonas aeruginosa* PAO1 infecting Hep2 epithelial cells. Artistic license allowed part of the Hep2 cell surface to become a pillow, and parts of the bacterial flagellum to morph into the sleepy eyes and mouth.

40 Tonney, F. O., Greeer, F. E and Danforth, T. F. 1928. The minimal “chlorine death points” of bacteria. *Am J Pub Health* **18**: 1259; Ridgway, H. F. and Olson, B. H. (1982) Chlorine resistance patterns of bacteria from two drinking water distribution systems. *Appl Environ Microbiol* **44**: 972–987; Behnke, S., Parker, A.E., Woodall, D. and Camper, A. K. (2011) Comparing the chlorine disinfection of detached biofilm clusters with those of sessile biofilms and planktonic cells in single‐ and dual‐species cultures. *Appl Environ Microbiol* **77**: 7176–7184; Behnke, S. and Camper, A. K. (2012) Chlorine dioxide disinfection of single and dual species biofilms, detached biofilm and planktonic cells. *Biofouling* **28**: 635–647.

41 https://pronunciationstudio.com/upper‐received‐pronunciation/

42
https://pronunciationstudio.com/upper-received-pronunciation

43 Fraile, S., Jiménez, J.I., Gutiérrez, C. and de Lorenzo, V. (2013) NanoPad: an integrated platform for bacterial production of camel nanobodies aimed at detecting environmental biomarkers. *Proteomics* **13:** 2766–2775; Pinero‐Lambea, C., Bodelon, G., Fernandez‐Perianez, R., Cuesta, A.M., Álvarez‐Vallinam L., and Fernández, L.Á. (2015) Programming controlled adhesion of *E. coli* to target surfaces, cells and tumours with synthetic adhesins. *ACS Synth Biol* **4:** 463–473; Glass, D.S. and Riedel‐Kruse, I.H. (2018) A synthetic bacterial cell‐cell adhesion toolbox for programming multicellular morphogenesis and patterns. *Cell* **174:** 649–658.

44 see, e.g. Timmis, K., Timmis, J., Brüssow, H., and Fernandez, L. A. (2019) Synthetic consortia of nanobody‐coupled and formatted bacteria for prophylaxis and therapy interventions targeting microbiome dysbiosis‐associated diseases and co‐morbidities. *Microbiol Biotechnol* **12:** 58‐65.

